# The Expression Profile and Textural Characteristics of C595-Reactive MUC1 in Pancreatic Ductal Adenocarcinoma for Targeted Radionuclide Therapy

**DOI:** 10.3390/cancers13010061

**Published:** 2020-12-28

**Authors:** Ashleigh Hull, Yanrui Li, Dylan Bartholomeusz, William Hsieh, Samantha Escarbe, Andrew Ruszkiewicz, Eva Bezak

**Affiliations:** 1 Cancer Research Institute and Allied Health and Human Performance Academic Unit, University of South Australia, Adelaide, SA 5000, Australia; judy.li@unisa.edu.au (Y.L.); william.hsieh@sa.gov.au (W.H.); eva.bezak@unisa.edu.au (E.B.); 2 Department of PET, Nuclear Medicine & Bone Densitometry, SA Medical Imaging, Royal Adelaide Hospital, Adelaide, SA 5000, Australia; dylan.bartholomeusz@sa.gov.au; 3 Adelaide Medical School, The University of Adelaide, Adelaide, SA 5000, Australia; 4 Centre for Cancer Biology, University of South Australia and SA Pathology, Adelaide, SA 5000, Australia; samantha.escarbe@sa.gov.au (S.E.); andrew.ruszkiewicz@sa.gov.au (A.R.); 5 Division of Anatomical Pathology, SA Pathology, Adelaide, SA 5000, Australia; 6 Department of Physics, The University of Adelaide, Adelaide, SA 5000, Australia

**Keywords:** pancreatic cancer, mucin 1, targeted radionuclide therapy, textural analysis

## Abstract

**Simple Summary:**

Pancreatic ductal adenocarcinoma (PDAC) is a cancer of low survival needing novel treatment approaches such as targeted therapies. If a target is overexpressed on PDAC cells but has minimal expression on normal cells, it is considered a good candidate for targeted therapy. Identifying targets with this expression pattern can help to optimise targeted therapies to be therapeutically effective without compromising on tolerability. The aim of this study was to assess the expression of the MUC1 receptor using the C595 antibody. We performed a series of cell line and tissue studies to identify if the expression of the MUC1 receptor changes between different pancreatic pathologies, including PDAC and normal pancreatic tissue. We found that the MUC1 receptor is both overexpressed and more uniformly expressed in PDAC compared to the other tissue types assessed. This indicates that the MUC1 receptor is a feasible target for targeted therapies of PDAC.

**Abstract:**

Improvements in the prognosis of pancreatic ductal adenocarcinoma (PDAC) rely on the development of effective treatments to target advanced disease. Mucin 1 (MUC1) is a transmembrane glycoprotein which is involved in the metastatic progression of PDAC and is a receptor-of-interest for targeted radionuclide therapy. The aim of this study was to determine the feasibility of MUC1-based targeted radionuclide therapy for PDAC, by evaluating the expression profile of MUC1 in different pancreatic cells and tissues using the C595 antibody. MUC1 expression was evaluated in four PDAC cell lines (PANC-1, BxPC-3, CAPAN-1 and AsPC-1) using flow cytometry and immunocytochemistry. Immunohistochemistry was performed on primary and metastatic PDAC, pancreatitis, pancreatic intra-epithelial neoplasia and normal pancreatic tissue samples to identify potential changes in C595-reactive MUC1 expression across different disease groups. C595-reactive MUC1 expression was found to varying degrees in the cell lines (11.5–93.1%). A pixel analysis of the immunohistochemical staining demonstrated highest MUC1 expression in primary PDAC tissue (mean pixel value of 205.4), followed by other pancreatic cancer types (204.9), pancreatic intra-epithelial neoplasia (203.8), metastatic PDAC (201.5), chronic pancreatitis (198.1) and normal pancreatic tissue (191.4). The increased expression in malignant tissues and reduced expression in benign tissues indicate that C595-reactive MUC1 is a potential target for targeted radionuclide therapy of PDAC.

## 1. Introduction

Pancreatic ductal adenocarcinoma (PDAC) is a highly aggressive malignancy with current five-year survival rates of only ~10% in Australia [1]. A lack of early detection methods and curative treatment options contribute to the poor prognosis of PDAC. Surgery remains the only curative PDAC treatment yet its application is limited to those with locally resectable disease only [2]. With 30–40% of newly diagnosed PDAC patients presenting with Stage III disease [3], therapies capable of controlling locally advanced and metastatic disease are needed.

Targeted radionuclide therapy (TRT) is a systemic therapy which uses molecular carriers to deliver highly cytotoxic radionuclides to cancerous cells. The high stability, minimal toxicity and highly selective targeting capacity has positioned monoclonal antibodies (mAbs) as the carrier of choice for TRT [4]. For effective TRT, the chosen mAb should ideally target an antigen or epitope that has increased and homogeneous expression on cancerous tissues compared to normal and benign tissues. Selection of an appropriate mAb and receptor allows for optimal tumour targeting with low radiation exposure to normal tissues, thus reducing the likelihood of TRT-induced side effects.

The cellular heterogeneity of PDAC cells complicates the identification of an effective TRT target [5,6]. The expression of a receptor within and between individual PDAC cells can vary significantly and has been linked to the development of treatment resistance [7]. Textural analysis, a field of radiomics, can be used to assess the homogeneity of receptor expression [8]. Textural descriptors, such as Haralick features, quantify the spatial distribution of pixels with the same intensity to represent the underlying tissue texture. A gray level co-occurrence matrix (GLCM), which defines the distribution of co-occurring neighbouring pixel values, can be used to calculate these descriptors [9]. Textural analysis is most commonly used to characterise tumour heterogeneity for prognostication and treatment selection [7].

**Figure 1:**
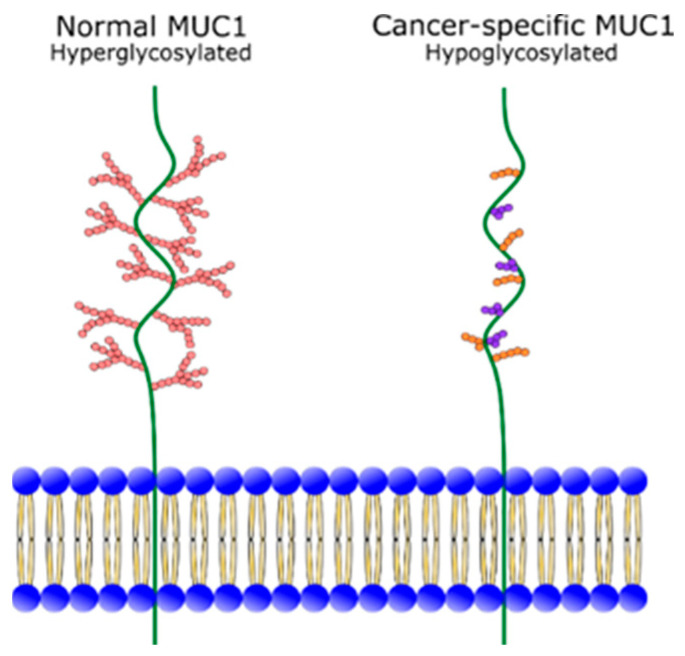
Glycosylation of normal MUC1 and cancer-specific MUC1. Image adapted from Roulois, et al. [18].

Mucin 1 (MUC1) is a transmembrane glycoprotein normally expressed on the apical surface of epithelial cells [10]. MUC1 consists of an extracellular domain, a transmembrane domain and a cytoplasmic tail, which extends intra-cellularly and has cell signalling functions [11,12]. The extracellular region of MUC1 is heavily O-glycosylated and extends 200–500 nm above the cell surface, to provide an anti-adhesive property, which physically protects the epithelial surface from pathogens [13,14,15]. A primary characteristic of the MUC1 extracellular domain is the variable number tandem repeat (VNTR) region within the peptide core. The VNTR region is comprised of 20–120 copies of 20–21 amino acid tandem repeats [13]. In normal cells, the VNTR region is concealed by the heavy glycosylation of the extracellular domain [14]. However, in epithelial cancers, such as PDAC, MUC1 is hypoglycosylated and loses apical polarisation (Figure 1) [10,16]. This exposes the VNTR region and leads to an aberrant overexpression and redistribution of MUC1 in cancer cells. The unmasking of the VNTR region gives different antigenic profiles between cancer-specific and physiological MUC1. Importantly for TRT, cancer-specific MUC1 epitopes (MUC1-CE) are revealed on cancerous tissues andare largely indiscernible on normal tissues that have physiologic MUC1 expression.

Cancer-specific MUC1 has a functional role in the progression of PDAC. MUC1 can disrupt and upregulate key pathways to facilitate the proliferation and metabolism of cancer cells. It also promotes angiogenesis, metastasis and chemoresistance through inactivation of the apoptotic pathways [14]. MUC1 levels have been positively correlated with more advanced PDAC [17]. As such, MUC1 is a favourable therapeutic and diagnostic target for PDAC.

C595 is an anti-MUC1 mAb which targets the Arg-Pro-Ala-Pro epitope repeatedly expressed on the VNTR region of the MUC1 peptide core [19,20]. Increased expression of C595-reactive MUC1 has been demonstrated in PDAC, breast and ovarian cancers [21,22,23]. Whilst C595 reacts with MUC1 on PDAC tissues, it is not clear when the C595-reactive VNTR region is exposed across the spectrum of pancreatic pathologies. To our knowledge, the homogeneity of C595-reactive MUC1 expression has also not been assessed in PDAC.

The aim of this study was to evaluate the expression profile and textural characteristics of C595-reactive MUC1 in PDAC cell lines, PDAC tissues, normal pancreas, pancreatitis and the PDAC precursor lesions, pancreatic intra-epithelial neoplasia (PanIN), to establish the feasibility of C595-based TRT for PDAC.

## 2. Results

### 2.1. Surface Expression of C595-Reactive MUC1 on Pancreatic Cancer Cells

**Figure 2:**
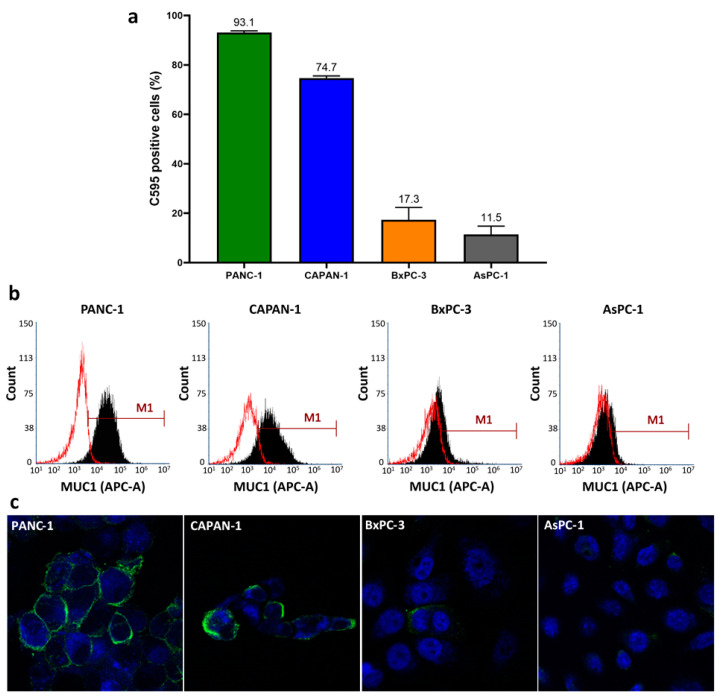
(**a**) Average surface expression of C595-reactive MUC1 on all four cell lines as determined by flow cytometry, (**b**) representative flow cytometry histograms demonstrating a positive shift (M1) of C595-reactive MUC1 (black) compared to isotype control (red) and (**c**) representative immunocytochemistry images at 63X magnification demonstrating varying levels of C595 staining on the surface of the analysed cells.

Surface expression of C595-reactive MUC1 was confirmed on all four cell lines (Figure 2). PANC-1 and CAPAN-1 demonstrated the greatest surface expression with an average of 93.1% and 74.7% of cells having positive MUC1 expression on flow cytometry. BxPC-3 and AsPc-1 cells had lower surface expressions of 17.3% and 11.5%, respectively. A similar pattern of MUC1 expression was noted by immunocytochemistry (Table 1), confirming the flow cytometry results.

### 2.2. Expression of C595-Positive MUC1 on Pancreatic Tissues

**Figure 3:**
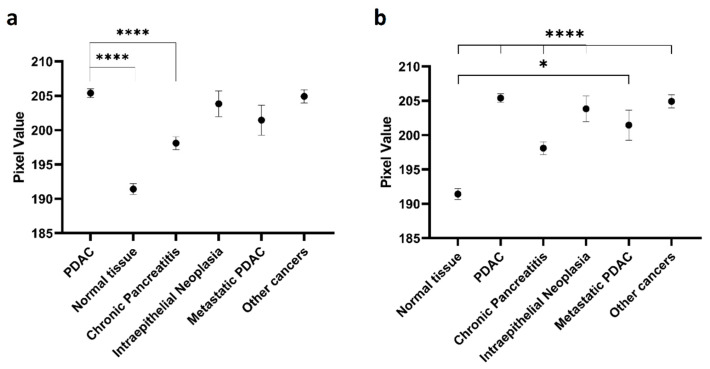
Mean pixel value and standard error measurement for different disease categories. (**a**) Significance compared to pancreatic ductal adenocarcinoma (PDAC) and **(b**) significance compared to normal tissue. * *p* < 0.05, **** *p* < 0.001.

Table 2 presents the demographic data of the analysed tissue samples. Immunohistochemical staining demonstrated increased expression on PDAC tissues compared to normal tissues. From the pixel analysis, PDAC had the greatest mean pixel value (205.4), closely followed by other pancreatic cancer types (204.9), PanIN (203.8) and metastatic PDAC (201.5) (Figure 3). Chronic pancreatitis had a mean pixel value of 198.1 whilst normal pancreatic tissue had a mean value of 191.4. The mean pixel values of normal and chronic pancreatitis tissues were significantly different to the mean pixel value of PDAC (*p* < 0.0001). There were no significant differences identified between PDAC and PanIN (*p* > 0.9999), PDAC and metastatic PDAC (*p* = 0.8822) and PDAC and other pancreatic cancers (*p* > 0.9999). All disease categories also significantly differed from normal pancreatic tissues (*p* < 0.05). These results are suggestive of increases in the expression of C595-reactive MUC1 as normal pancreatic tissue transitions to PDAC. Table 3 summarises the results of the significance testing.

### 2.3. Validation of Pixel Analysis

The histopathologist scoring supports the pixel analysis results. Evaluating both the staining intensity and percentage of stained cells, the histopathologist scoring demonstrated increased staining on the PDAC tissue samples compared to normal tissues (Table 4). Metastatic PDAC and chronic pancreatitis samples had similar immunoreactive scores to PDAC. Mean pixel values for the subset of 25 tissue samples ranged from 200.5 (normal tissue) to 204.4 (primary PDAC), suggesting MUC1 expression was more similar in this subset compared to the full tissue sample.

**Figure 4:**
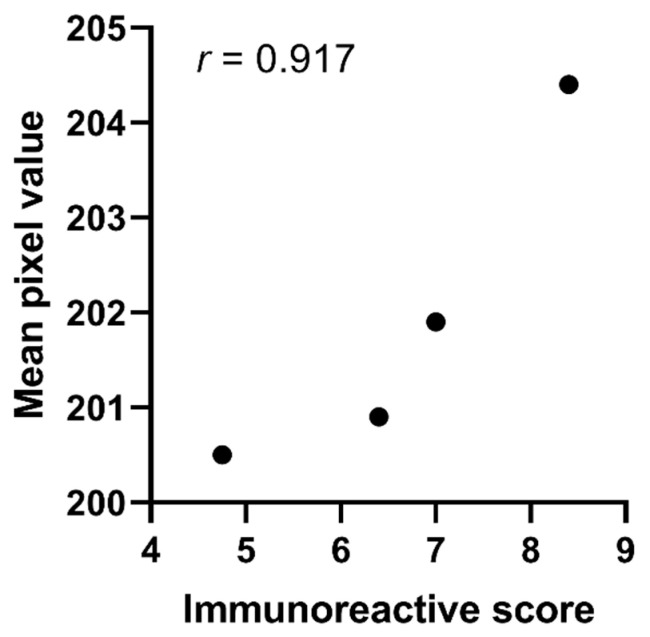
Histopathologist validation of the mean pixel value through correlation to mean immunoreactive score for a subset of 25 tissue samples.

A strong linear relationship (*r* = 0.917) was identified between the mean pixel value and the average immunoreactive score using Pearson’s correlation co-efficient (Figure 4). The relationship between mean pixel value and average immunoreactive score was found to not be significant (*p* = 0.083), suggesting that pixel analysis is a valid measure to assess immunohistochemical staining.

### 2.4. Textural Analysis of Pancreatic Tissues

In total, 15 textural descriptors consisting of Haralick features and kurtosis values were calculated for the analysed immunohistochemistry tissues. The median values and 95% confidence intervals for each disease category are displayed in Table 5.

**Figure 5:**
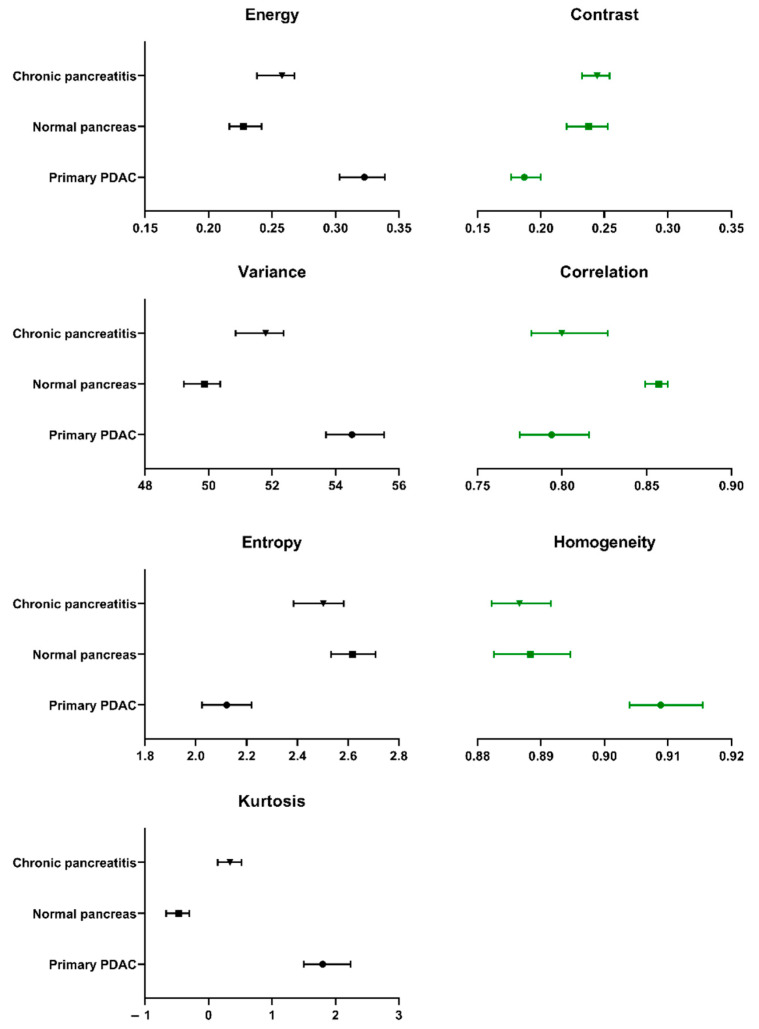
Median values and 95% confidence intervals for the textural descriptors (Haralick features and kurtosis) used to analyse PDAC, chronic pancreatitis and normal pancreatic tissue samples.

Due to the uneven sample sizes, significance testing was only performed on PDAC, normal pancreas and chronic pancreatitis samples (Table 6). Significant differences were identified for all 15 textural descriptors between PDAC and normal pancreatic tissues. There were also significant differences between chronic pancreatitis and PDAC for all analysed descriptors, with the exception of correlation and information measure of correlation I. PDAC tissues demonstrated the greatest textural uniformity (energy) and homogeneity, yet also had the greatest dispersion of pixel values (variance) and were more outlier-prone (kurtosis) compared to the other assessed groups. Comparatively, normal tissues were more heterogeneous, with a greater degree of randomness (entropy). Normal tissue also demonstrated the greatest degree of linear dependency, or correlation, between neighbouring pixel values. The differences between the primary textural descriptors of PDAC, normal pancreas and chronic pancreatitis are represented in Figure 5. Further textural descriptors, derived from the primary descriptors, are presented in Supplementary Materials Figure S1.

## 3. Discussion

An inability to treat local and systemically advanced disease is a contributing factor to the poor survival rates of PDAC. TRT remains a potential therapeutic option for PDAC however, to reach clinical relevance, appropriate target identification and treatment stratification processes are needed [24]. In this study, we evaluated the textural characteristics and expression of C595-reactive MUC1 across different immunostained pancreatic tissues and cells. The expression of C595-reactive MUC1 has been previously investigated by Qu et al. [21], who demonstrated strong expression in PDAC tissues and weak expression in normal tissues. We aimed to extend this knowledge by also assessing C595-reactive MUC1 expression in benign pancreatic conditions such as pancreatitis and PanIN. We have confirmed the C595-reactive MUC1 expression changes throughout different pancreatic conditions. In the current study, MUC1 expression was strongest in malignant pancreatic tissues including all analysed pancreatic cancer types. These findings suggest MUC1 glycosylation changes may occur prior to full PDAC transformation, with C595-based therapies capable of targeting and impairing both early and late stage disease.

Surface expression of four pancreatic cancer cell lines was also identified by flow cytometry and immunocytochemistry. Surface expression was greatest on the primary PDAC cell line, PANC-1, and CAPAN-1, a cell line developed from a PDAC liver metastasis. The percentage of C595-positive CAPAN-1 cells was lower in the current study (74%) compared to previously reported results (95%) [21]. Variability in the MUC1 expression was also noted in BxPC-3, a primary PDAC cell line, and AsPC-1, a cell line derived from ascites metastases. These results suggest PDAC cells have heterogeneous C595-reactive MUC1 surface expression which can complicate targeted therapy development. However, as PANC-1 and CAPAN-1 demonstrated strong MUC1 expression, C595-based targeted therapies may still be beneficial when directed towards subpopulations of PDAC cells with positive MUC1 expression. 

Several anti-MUC1 antibodies have already been investigated for PDAC therapy and diagnosis. To date, the most successful TRT was 90Y-PAM4, which progressed to clinical trials [25,26]. Unfortunately these trials were prematurely terminated [27]. Other anti-MUC1 antibodies have included TAB004 [28], CT2 [29], DF3 [30], AR20.5 [31] and MA5 [32], although these have not all been assessed as TRT agents. Specificity for pancreatic cancer has been a key contributor to the progression of PAM4, TAB0004 and AR20.5 antibodies. Our study demonstrated low levels of MUC1 expression on normal pancreatic tissue, suggesting the C595 mAb is not 100% specific for PDAC. However, minimal expression on normal pancreatic tissues may be overcome by balancing treatment regimes and considering fractionated TRT schedules, which allow for normal tissue repair and cancer cell radiosensitisation in between treatment delivery [33]. For TRT, the outcomes affected by normal tissue expression would also be impacted by choice of beta- or alpha-emitting radionuclide. Alpha-emitting radionuclides, such as actinium-225, have a higher cytotoxicity and are more damaging to targeted tissues than beta-emitting radionuclides [34]. Consequently, target expression on normal tissues may have greater implications for targeted alpha therapy than targeted beta therapy. This is demonstrated by prostate-specific membrane antigen (PSMA)-based TRT of prostate cancer. PSMA is overexpressed on approximately 80% of prostate cancer cells and physiologically expressed by the salivary glands [35]. Despite cancerous cells exhibiting 100- to 1000-fold greater PSMA expression, the normal tissue expression in the salivary glands has led to xerostomia in patients receiving PSMA-TRT [36]. The severity of xerostomia ranges from mild to moderate for lutetium-177 PSMA-TRT [37] yet it is a severe dose-limiting toxicity in actinium-225 PSMA-TRT [38]. Additional strategies capable of overcoming normal tissue expression are therefore needed to optimise tolerability and therapeutic efficacy for targeted alpha therapy.

Tumour heterogeneity often complicates the effectiveness of targeted therapies. Heterogeneity within tumours typically arises from angiogenesis, hypoxia-induced changes and genomic expression and can reflect subclonal tumour populations [39,40]. Textural analysis can be used to characterise tumour heterogeneity, with studies demonstrating its value in prognostication and treatment selection for PDAC [7,39,40,41]. Whilst textural analysis is mostly used in the assessment of medical images, we extended the analysis to evaluate the homogeneity of MUC1 receptor expression in C595 immunostained pancreatic tissues. The current study demonstrated significant differences between the textural descriptors of PDAC, normal tissues and chronic pancreatitis. PDAC tissues were texturally uniform, demonstrating more homogenous expression of C595-reactive MUC1 compared to normal pancreatic and chronic pancreatitis tissues. PDAC tissue was also the most outlier-prone, with respect to the kurtosis. A recent study investigating textural analysis of computed tomography images showed high kurtosis was significantly correlated with worse prognosis in PDAC patients, thus the kurtosis measurement may be valuable for prognostication [39]. Chronic pancreatitis and normal pancreatic tissue demonstrated similar textural trends. It would be expected that the inflammatory nature of chronic pancreatitis would lead to textural heterogeneity in affected tissues. This trend was not effectively observed in the current study and may suggest C595 staining counteracted the inflammation-induced textural changes in chronic pancreatitis. Further studies should consider full characterisation of the textural features of the pancreatic disease spectrum. Textural analysis of receptor expression may be a valuable method for treatment selection and should be considered in the development of TRT. The overall value of textural analysis and its application to prognostication and treatment selection for C595-based targeted therapies will be clearer in future animal studies and clinical trials. Extension into artificial intelligence systems is also possible [42].

## 4. Materials and Methods

### 4.1. Antibodies

The C595 mAb was purchased from Medical Scitec Australia Pty Ltd. (Sydney, Australia) and prepared by QED Bioscience (San Diego, CA, USA). An irrelevant mouse IgG3 isotype control antibody was purchased from Abcam (Cambridge, UK). Alexa Fluor 488 goat anti-mouse IgG and Alexa Fluor 647 goat anti-mouse IgG secondary antibodies were purchased from Thermo Fisher Scientific Australia Pty Ltd. (Scoresby, Australia). Biotinylated goat anti-mouse IgG (BD Pharmingen, San Diego, CA, USA) was kindly supplied by S.E.

### 4.2. Cell Cultures

Four human pancreatic cancer cell lines (BxPC-3, PANC-1, CAPAN-1 and AsPC-1) were purchased from American Type Culture Collection (Manassas, VI, USA) via In Vitro Technologies Pty Ltd. (Noble Park North, Australia). BxPC-3 and AsPC-1 cells were cultured using Roswell Park Memorial Institute (RPMI) 1640 medium (Sigma-Aldrich Fine Chemicals, St Louis, MO, USA) supplemented with 10% foetal bovine serum (FBS) and 1% penicillin/streptomycin (P/S). CAPAN-1 cells were cultured using Iscove’s Modified Dulbecco’s Medium (IMDM) (Sigma-Aldrich Fine Chemicals, St Louis, MO, USA) supplemented with 20% FBS and 1% penicillin/streptomycin. PANC-1 cells were cultured using Dulbecco’s Modified Eagle Medium (DMEM) (Sigma-Aldrich, Castle Hill, NSW, Australia) supplemented with 10% FBS and 1% P/S. All cell lines were grown in T75 flasks and incubated at 37 °C in a 5% carbon dioxide in air atmosphere. Cells were routinely assessed for mycoplasma contamination and used within three months of receipt or resuscitation. For experiments, cells were grown to confluence then washed twice with phosphate-buffered saline (PBS) and detached from the flask by TrypLE Select Enzyme (1X) (Thermo Fisher Scientific Australia Pty Ltd., Scoresby, Australia). All cell culture reagents and culture media were purchased from Sigma-Aldrich Pty Ltd. (Castle Hill, Australia) unless otherwise stated.

### 4.3. Flow Cytometry

To detect surface expression of C595-reactive MUC1, an indirect immunofluorescence staining procedure was performed on the four cell lines. Approximately 2.0 × 10^5^ cells were harvested and added to flow cytometry tubes. The cells were centrifuged at 300× *g* for 5 min to separate the cell pellet and supernatant. The supernatant was removed from each tube. Cells were then washed twice in a buffer consisting of 1% FBS diluted in Dulbecco’s PBS (DPBS) (Sigma-Aldrich, Castle Hill, NSW, Australia). For each wash, cells were centrifuged at 300× *g* for 4 min before the buffer was replaced. After the second wash, cells were resuspended in 1% FBS/DPBS. Cells were then incubated on ice with C595 (10 µg/mL, 100 µL) or isotype control (5 µg/mL, 100 µL) for 30 min. Following incubation, cells were washed and resuspended in 1% FBS/DPBS using the procedure outlined above. Cells were then incubated with Alexa Fluor 647 goat anti-mouse secondary antibody (Thermo Fisher Scientific, Waltham, MA, USA) (4 µg/mL, 100 µL) for 30 min on ice in a dark cupboard. A final wash series was performed and cells were resuspended in 1% FBS/DPBS. A BD Accuri C6 Plus Flow Cytometer (Becton Dickinson, New Jersey, NJ, USA) was used to analyse the cells. For each cell line, triplicate samples of C595, isotype control, propidium iodide (PI) and unstained cells were analysed. Flow cytometry data was processed using FCS Express 7 Software (De Novo Software, Pasadena, CA, USA).

### 4.4. Immunocytochemistry

Cells (2.5 × 10^5^) suspended in 500 µL of media were grown on glass coverslips in a 24-well plate overnight at 37 °C. The following day, media was removed from the wells and cells were washed twice using PBS. Cells were then fixed to the coverslips by incubating with 10% formaldehyde for 7.5 min at room temperature. Formaldehyde was removed and cells were washed twice using PBS. Non-specific antibody binding was blocked by incubating cells in 5% bovine serum albumin (BSA)/PBS solution at room temperature for 45 min. Excess BSA/PBS was removed before cells were washed twice using PBS. The coverslips were then placed cell-side down on antibody droplets of either C595 (5 µg/mL, 100 µL) or the isotype control (2.5 µg/mL, 100 µL) in an incubation chamber and incubated overnight at 4 °C. The following day, the coverslips were placed into the well plate and washed three times using PBS. For each wash, the well plate was placed on a shaking table set at 70 rpm for 5 min. Coverslips were then placed cell-side down on droplets of Alexa Fluor 488 goat anti-mouse secondary antibody (4 µg/mL, 100 µL) and incubated for 1 h at room temperature in a dark cupboard. Following incubation, cells were washed three times on the shaking table using PBS. Cells were then counterstained with DAPI (4′,6-diamidino-2-phenylindole, 0.5 µg/mL, 100–300 µL) and incubated at room temperature for 5 min. Cells were then washed again. Coverslips were mounted onto microscope slides using Fluoroshield with 1,4-Diazabicyclo[2.2.2]octane (Sigma-Aldrich Pty Limited, Castle Hill, Australia). A ZEISS LSM 800 confocal microscope (ZEISS, Oberkochen, Germany) was used to acquire images of the cells. Images were visually assessed using Image J (National Institutes of Health, Bethesda, MD, USA) and characterised as focal or diffuse staining. A staining intensity score of 0–3 (0: no staining, 1: weak, 2: moderate, 3: strong staining) was also assigned for each cell type.

### 4.5. Human Pancreatic Tissues

Four unstained human paraffin-embedded tissue microarrays (TMA) (PA2081c, PA485, HPanA060CS02 and BIC14011b) were purchased from US Biomax (Rockville, USA). The TMA included tissue samples of primary and metastatic PDAC, normal pancreas, acute and chronic pancreatitis, PanIN and other pancreatic cancers (e.g., neuroendocrine tumours). Both sexes were represented across the tissue samples. The TMAs were processed for immunohistochemical staining.

### 4.6. Immunohistochemical Staining

TMAs were initially deparaffinised using xylene then rehydrated with 100% ethanol and washed with PBS. A heat-mediated antigen retrieval process was then performed using a fresh citrate buffer (pH 6.5). Endogenous peroxidase activity was quenched by incubating TMAs in 1% hydrogen peroxide for 10 min. TMAs then underwent four washes (3 × distilled water, 1 × PBS). Non-specific binding was blocked by incubating TMAs with 10% normal goat blocking serum at room temperature for 60 min. Excess serum was removed from the tissues by blotting. TMAs were incubated overnight with C595 antibody (13 µg/mL) diluted in 3% normal serum at 4 °C. The following day, tissues were washed three times with PBS and incubated with a biotinylated goat anti-mouse antibody (2 µg/mL) diluted in 3% normal serum for 35 min. After incubation, TMAs were washed three times using PBS then incubated with VECTASTAIN elite ABC reagent (PK-6100, Vector Laboratories, Burlingame, CA, USA). TMAs were washed again using PBS and incubated in a DAB peroxidase solution (Cat#Sk-4100, Vector Laboratories, Burlingame, CA, USA) until desired stain intensity was obtained. TMAs were rinsed in tap water and counterstained with haemotoxylin (DAKO, Glostrup, Denmark). Then, TMAs were rinsed with tap water, dipped in acid alcohol, rinsed with tap water and 70% ethanol, washed twice with 100% ethanol and once with xylene. TMAs were then mounted onto microscope slides using Fluoroshield. All TMAs were imaged using a NanoZoomer 2.0-HT Digital Slide Scanner (Hamamatsu Photonics, Hamamatsu City, Japan), courtesy of Adelaide Microscopy.

### 4.7. Analysis of Immunohistochemical Staining

#### 4.7.1. Pixel Analysis

All TMA images were reviewed and cropped to individual tissue sections using NDP.view software (v.2.6.13, Hamamatsu Photonics, Hamamatsu City, Japan). The individual tissue images were analysed using Image J (v.1.52, National Institutes of Health, Bethesda, MD, USA). Unnecessary background signal was removed by colour thresholding (background signal was assigned a pixel value of 0). Total pixel count and histograms containing the distribution of pixel values (0–256) were produced for each image. The histogram and total pixel count were background-corrected by subtracting the count at the pixel value of 0. Descriptive statistics including the mean, standard deviation, minimum and maximum pixel values were then calculated using the background-corrected histogram. All background correction processes were performed using MATLAB (v.2020a, MathWorks, Natick, MA, USA). The background-corrected mean pixel values and standard error measurements were averaged according to disease type and plotted using GraphPad Prism (v.8.2.0, GraphPad Software Inc, San Diego, CA, USA). To evaluate for significant differences between the mean pixel values of the disease categories, a Kruskal–Wallis Test was performed. Two post-hoc Dunn’s multiple comparison tests were also performed using PDAC and normal tissues as the controls, respectively. Non-parametric analyses were used as the data between the disease categories was not considered normally distributed.

#### 4.7.2. Validation of Pixel Analysis

To validate the pixel analysis, a sample of 25 tissue sections consisting of primary PDAC (*n* = 5), metastatic PDAC (*n* = 5), PanIN (*n* = 5), pancreatitis (*n* = 5) and normal pancreatic tissue (*n* = 5) were scored by an experienced histopathologist. For each tissue sample, a score from 0 to 4 was assigned for the percentage of MUC1-positive stained cells (0: no staining, 1: 1–10%, 2: 11–50%, 3: 51–80%, 4: 81–100% of cells stained) and a score from 0 to 3 was assigned to grade the intensity of the staining (0: no staining, 1: weak, 2: moderate, 3: strong staining). The final immunoreactive score was calculated as the product of the two scores. The maximum immunoreactive score was 12. The average immunoreactive score was calculated according to disease category and correlated with the mean pixel value using Pearson’s correlation co-efficient.

#### 4.7.3. Textural Analysis

As a final analysis, the texture of the immunohistochemically stained tissue was also analysed using Haralick features and kurtosis measurements. Initially, individual tissue images obtained from the NanoZoomer were converted to 8-bit grayscale using Image J. The 14 Haralick features were then calculated for each image using MATLAB, with a code modified from Monzel [43]. Briefly, MATLAB processing involved the calculation of a GLCM. Each Haralick feature was then calculated using the equations originally described by Haralick, et al. [44]. The average values for each Haralick feature were calculated according to disease groups. Kurtosis values were also calculated using Equation (1):

(1) $$Kurtosis=\;\frac{E\left(x-\mu \right)4}{\sigma 4}-3$$

where *μ* is the mean of *x*, *σ* is the standard deviation of *x*, and *E*(*t*) represents the expected value of the quantity *t*.

The value of 3 was subtracted to provide a normal distribution with a kurtosis of 0. Significant differences between the average Haralick feature values of PDAC and normal pancreatic tissue, and PDAC and chronic pancreatitis tissue, were determined using the Mann–Whitney test. The Mann–Whitney test was selected as the textural data was non-parametric and independent between disease groups. As the power of the Mann–Whitney test decreases as sample sizes become more unequal, significance testing was only performed on groups with similar sample size. For this reason, significance testing was performed on only PDAC (*n* = 106), chronic pancreatitis (*n* = 77) and normal pancreatic tissue (*n* = 97) samples.

## 5. Conclusions

Our study demonstrated C595-reactive MUC1 expression in PDAC, with significantly lower expression in normal and chronic pancreatitis tissues. Further work exploring methods to reduce damage to these normal tissues is needed. MUC1 expression also appears more homogeneous in PDAC tissues, indicating a greater potential therapeutic effect for malignant tissues with reduced exposure to benign tissues. These results are supportive of future investigations into the development of C595-based targeted radionuclide therapies for PDAC.

## Figures and Tables

**Table 1:** Staining intensity and pattern of cell lines following immunocytochemistry.

Cell Line	Staining Intensity ^1^	Staining Pattern
PANC-1	3	Diffuse
CAPAN-1	2	Focal
BxPC-3	1	Diffuse
AsPC-1	1	Focal

^1^ Staining intensity score ranged from 0 to 3 (0: no staining, 1: weak, 2: moderate, 3: strong staining).

**Table 2:** Demographic data of analysed tissue samples according to disease type.

Disease Category	Sample Size (*n*)	Median Age (Range)
All samples	357	54 (1083)
Normal pancreatic tissue	97	54 (21–76)
Pancreatic ductal adenocarcinoma	106	58 (23–83)
Metastatic pancreatic ductal adenocarcinoma	10	59 (51–66)
Acute pancreatitis	7	60 (47–68)
Chronic pancreatitis	77	55 (10–76)
Pancreatic intraepithelial neoplasia	22	52 (36–67)
Other cancer	38	48 (17–77)

**Table 3:** Significance testing of the mean pixel value using Dunn’s multiple comparison test.

Disease Category	*p*-Values Compared to Control Tissue	
Test Tissues	PDAC	Normal Pancreatic Tissue
Pancreatic ductal adenocarcinoma	-	<0.0001 *
Normal tissue	<0.0001 *	-
Chronic pancreatitis	<0.0001 *	<0.0001 *
Pancreatic intraepithelial neoplasia	>0.9999	<0.0001 *
Metastatic PDAC	0.8882	0.0110 *
Other pancreatic cancers	>0.9999	<0.0001 *

* *p* < 0.05.

**Table 4:** Average histopathology scores and mean pixel value according to disease category for subset of analysed tissues.

Disease Category	Average Score Assigned by Histopathologist		Average Immunoreactive Score ^3^	Mean Pixel Value
	Percentage of Stained Cells ^1^	Intensity of Staining ^2^		
Primary PDAC	3.4	2.4	8.4	204.4
Metastatic PDAC	3.5	2.3	8.0	200.9
Chronic Pancreatitis	3.2	2.0	7.4	201.9
Normal Pancreas	2.8	1.6	4.8	200.5

^1^ Percentage of stained cells’ scores ranged from 0 (no stained cells) to 4 (81–100% of stained cells); ^2^ intensity of staining scores ranged from 0 (no staining) to 3 (strong staining). ^3^ The immunoreactive score is the product of the percentage of stained cells and staining intensity scores.

**Table 5:** Median values and 95% confidence intervals for the textural descriptors.

Textural Descriptor	PDAC			Normal Pancreas			Metastatic PDAC			Chronic Pancreatitis			PanIN		
	Median	95% CI		Median	95% CI		Median	95% CI		Median	95% CI		Median	95% CI	
		L	U		L	U		L	U		L	U		L	U
Sample size (*n*)	106			97			10			77			22		
Energy	0.3228	0.3031	0.3390	0.2273	0.2165	0.2418	0.2523	0.2308	0.3159	0.2580	0.2380	0.2676	0.2935	0.2579	0.3270
Contrast	0.1870	0.1766	0.199	0.2377	0.2204	0.2527	0.1988	0.1578	0.2155	0.2443	0.2324	0.2542	0.2241	0.1988	0.2348
Correlation	0.7939	0.7751	0.8159	0.8572	0.8492	0.8624	0.8446	0.7453	0.8741	0.7999	0.7818	0.8270	0.7853	0.7323	0.8189
Variance	54.52	53.70	55.55	49.87	49.23	50.37	51.32	50.68	54.98	51.80	50.85	52.37	53.31	51.89	55.27
Homogeneity	0.9089	0.9039	0.9155	0.8884	0.8826	0.8946	0.9035	0.8972	0.9224	0.8866	0.8823	0.8916	0.8948	0.8894	0.9061
Sum Average	14.73	14.60	14.88	14.02	13.95	14.09	14.27	14.14	14.82	14.35	14.21	14.42	14.57	14.37	14.85
Sum Variance	180.8	178.2	187.0	156.5	154.1	159.1	164.5	158.7	184.7	166.3	162.5	169.6	174.7	168.0	186.0
Sum Entropy	1.337	1.272	1.380	1.639	1.601	1.684	1.510	1.268	1.662	1.530	1.481	1.587	1.383	1.272	1.516
Entropy	2.122	2.025	2.219	2.617	2.533	2.709	2.371	2.060	2.633	2.502	2.385	2.583	2.212	2.038	2.420
Difference Variance	0.1580	0.1497	0.1669	0.1970	0.1835	0.2087	0.1659	0.1345	0.1793	0.2032	0.1927	0.2092	0.1853	0.1661	0.1970
Difference Entropy	0.4828	0.4678	0.5027	0.5526	0.5312	0.5721	0.5010	0.4370	0.5248	0.5612	0.5469	0.5742	0.5362	0.5013	0.5464
Information Measure of Correlation I	−0.4427	−0.4737	−0.4253	−0.4996	−0.5174	−0.4787	−0.5072	−0.5671	−0.3923	−0.4452	−0.4738	−0.4378	−0.4367	−0.4979	−0.3747
Information Measure of Correlation II	0.9979	0.9973	0.9983	0.9996	0.9995	0.9996	0.9992	0.9971	0.9996	0.9991	0.9989	0.9994	0.9983	0.9970	0.9991
Maximal Correlation Coefficient	0.8169	0.7951	0.8287	0.8795	0.8694	0.8902	0.8605	0.7599	0.8930	0.8387	0.8196	0.8616	0.8144	0.7437	0.8709
Kurtosis	1.802	1.498	2.244	−0.4732	−0.666	−0.3023	0.4964	−0.6286	1.192	0.3385	0.1463	0.5188	0.7882	−0.06413	1.767

95% CI: 95% confidence interval of median, L: lower limit, U: upper limit.

**Table 6:** Significance testing of the textural descriptors between PDAC and normal pancreatic tissue, and PDAC and chronic pancreatitis tissues.

Textural Descriptor	Normal Tissue	Chronic Pancreatitis
	*p*-Values Compared to PDAC Tissues (* *p* < 0.05)	
Energy	<0.0001 *	<0.0001 *
Contrast	<0.0001 *	<0.0001 *
Correlation	<0.0001 *	0.3614
Variance	<0.0001 *	<0.0001 *
Homogeneity	<0.0001 *	<0.0001 *
Sum Average	<0.0001 *	<0.0001 *
Sum Variance	<0.0001 *	<0.0001 *
Sum Entropy	<0.0001 *	<0.0001 *
Entropy	<0.0001 *	<0.0001 *
Difference Variance	<0.0001 *	<0.0001 *
Difference Entropy	<0.0001 *	<0.0001 *
Information Measure of Correlation I	0.0006 *	0.7915
Information Measure of Correlation II	<0.0001 *	<0.0001 *
Maximal Correlation Coefficient	<0.0001 *	0.0230 *
Kurtosis	<0.0001 *	<0.0001 *

## Data Availability

The data presented in this study are available on request from the corresponding author, due to privacy concerns.

## Supplementary Materials

The following are available online at https://www.mdpi.com/2072-6694/13/1/61/s1, Figure S1: Median and 95% confidence intervals for derived Haralick features for PDAC, chronic pancreatitis and normal pancreas tissue samples.
