# Supplementary material for: The Expression Profile and Textural Characteristics of C595-Reactive MUC1 in Pancreatic Ductal Adenocarcinoma for Targeted Radionuclide Therapy
Source: Cancers (Basel). 2020 Dec 28;13(1):61. doi: 10.3390/cancers13010061 (PMC7796161; doi:10.3390/cancers13010061)
Supplement: Supplementary file 1 [file cancers-13-00061-s001.pdf]

Supplementary Material: The Expression Profile and Textural Characteristics of C595-Reactive MUC1 in Pancreatic Ductal Adenocarcinoma for Targeted Radionuclide Therapy

Ashleigh Hull, Yanrui Li, Dylan Bartholomeusz, William Hsieh, Samantha Escarbe, Andrew Ruszkiewicz and Eva Bezak

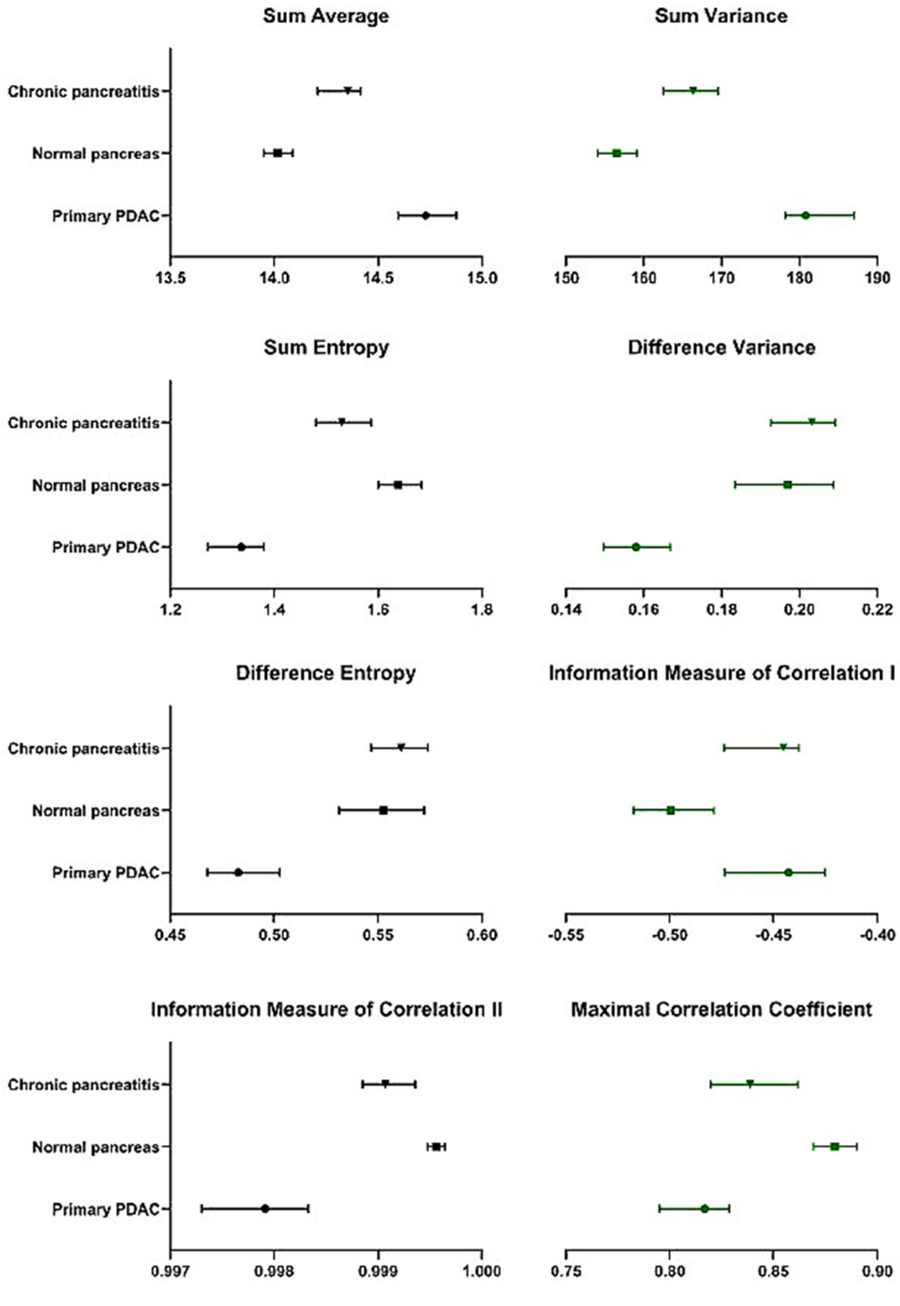

Figure S1. Median and 95% confidence intervals for derived Haralick features for PDAC, chronic pancreatitis and normal pancreas tissue samples.
